# Arsenic, Oxidative Stress and Reproductive System

**DOI:** 10.3390/jox12030016

**Published:** 2022-07-18

**Authors:** Felor Zargari, Md. Shiblur Rahaman, Robab KazemPour, Mahbobeh Hajirostamlou

**Affiliations:** 1 Department of Medical Science, Marand Branch, Islamic Azad University, Marand 5418916571, Iran; 2 Department of Environmental Science and Disaster Management, Noakhali Science and Technology University, Noakhali 3814, Bangladesh; shiblu.ju@gmail.com; 3 Department of Midwifery, Marand Branch, Islamic Azad University, Marand 5418916571, Iran; r_kazempour@marandiau.ac.ir; 4 Department of Biology, Marand Branch, Islamic Azad University, Marand 5418916571, Iran; hajirostamlou@marandiau.ac.ir

**Keywords:** arsenic, oxidative stress, male infertility, female infertility, toxicity

## Abstract

Infertility is a severe medical problem and is considered a serious global public health issue affecting a large proportion of humanity. Oxidative stress is one of the most crucial factors involved in infertility. Recent studies indicate that the overproduction of reactive oxygen species (ROS) or reactive nitrogen species (RNS) may cause damage to the male and female reproductive systems leading to infertility. Low amounts of ROS and RNS are essential for the normal functioning of the male and female reproductive systems, such as sperm motility, acrosome reaction, interactions with oocytes, ovulation, and the maturation of follicles. Environmental factors such as heavy metals can cause reproductive dysfunction in men and women through the overproduction of ROS and RNS. It is suggested that oxidative stress caused by arsenic is associated with male and female reproductive disorders such as through the alteration in sperm counts and motility, decreased sex hormones, dysfunction of the testis and ovary, as well as damage to the processes of spermatogenesis and oogenesis. This review paper highlights the relationship between arsenic-induced oxidative stress and the prevalence of infertility, with detailed explanations of potential underlying mechanisms.

## 1. Introduction

Infertility is not being able to become pregnant after one year or more of engaging in unprotected sex. Infertility affects millions of people of reproductive age worldwide and based on World Health Organization (WHO) reports, it is suggested that between 48 million couples and 186 million individuals suffer from infertility globally [1]. Medical studies have reported that 30% of infertile couples have unexplained infertility conditions after standard evaluations [2]. There are numerous reasons for the development of infertility in humans, and exposure to toxic heavy metals is considered one of the potential reasons. Humans are often exposed to individual or multiple heavy metals released from industrial activities into the environment. Within the last century, there has been a rapid growth in industrialization and technological advances across the world, which have contributed significantly to industrial pollution. Heavy metal pollution (i.e., arsenic, cadmium, mercury, titanium, iron, cobalt) is one of the industrial pollutions that is most prevalent in the environment. Arsenic is a well-known ubiquitous metalloid in the environment. Humans are mainly exposed to arsenic via contaminated drinking water and food. Arsenic is widely used in herbicides and insecticides for its known toxicological outcomes in invertebrates. Manifestations of arsenic toxicity in humans mainly depend on two factors: (I) the duration of the exposure and (II) the dose of the metalloid. Side effects associated with arsenic doses vary from fatality to cancers of the skin, bladder, liver, and kidney. Arsenic exposure has also contributed significantly to infertility and miscarriages in humans [3,4,5]. Findings from several epidemiological studies on menstruation indicate that exposure to heavy metals affects hormone levels in the human system [6]. There is very strong evidence that exposure to environmental levels of heavy metals interfere with the reproductive function in adult women [6,7]. Several epidemiological studies have evaluated blood levels of arsenic and other heavy metals in infertile women; among them, a notable investigation was conducted in China by Hsiao-Ling Lei et al., and the results showed that blood levels of arsenic were significantly higher in infertile women compared to pregnant women [8]. Several urinary biomarkers such as acylcarnitines, uridine (a stimulant of energy expenditure and apoptosis), and methyl xanthine have shown an association between arsenic-induced oxidative stress and infertility in men [9]. Numerous animal and human studies have shown that arsenic exposures induce infertility by altering the levels of follicle-stimulating hormone (FSH), luteinizing hormone (LH), and testosterone and may cause damage to the process of spermatogenesis and oogenesis [5,9]. Decreases in the fusion of egg–sperm [9], abnormalities in the shape of the sperm [6], disturbances of sperm capacitation [10], impairments in the glycolysis pathway [11], as well as disruptions in lipid and amino acids’ metabolism [12], are known adverse outcomes of arsenic for the male reproductive system. This review highlights the relationship between arsenic-induced oxidative stress and infertility.

## 2. Oxidative Stress

Oxidative Stress (OS) occurs as a result of an imbalance between ROS production and the antioxidant defense system in the cell; this causes deleterious effects on the cell’s life [13,14]. OS causes damage to the cell structure, lipid, protein, DNA, membrane of the cell, and cell signaling, and leads to several disorders such as diabetes [13], atherosclerosis [14], cancer [15], and infertility [16]. ROS are produced by oxygen metabolism. Enzymatic sources of ROS are monoamine oxidase (MAO), NAD(P)H oxidase, xanthine oxidase (XO), cyclooxygenase (COX), lipoxygenase (LOX), myeloperoxidase (MPO), and nitric oxide synthase (NOS) [14,17]. Electron transport chains in mitochondria, smoking, industrial waste products, viral and bacterial infection (phagocytic cells), environmental pollutants, and various cellular pathways such as protein tyrosine kinase and nuclear factor kappa B (NF-kB) represent alternate sources of ROS production. Small amounts of ROS are useful for signaling pathways such as protein tyrosine kinase, NF-kB, the G-protein-coupled receptor, gene expression, proliferation, ovulation, maturation of the follicle, and folliculogenesis [18,19,20,21,22]. Nitric oxide (NO) has an important role in folliculogenesis. The overproduction of NO has been observed in infertile women and men with varicocele. Common forms of ROS that affect sperm survival and its function include H_2_O_2_, OH• [23,24], peroxyl radicals, and hypochlorite ions [25,26]. ROS-induced damage to carbohydrates and lipids leads to the formation of ketoamines and ketoaldehydes [27], alkyl radicals, such as malondialdehyde (MDA), and 4-hydroxynonenal (HNE) (i.e., biomarkers of lipid peroxidation), respectively [28]. The oxidation of proteins by ROS leads to the production of modified proteins, alteration of their electrical charges, cleavages of the polypeptide chain, and formation of carbonyl groups (i.e., markers of protein oxidation) [27]. Breaking of the DNA strands, oxidized bases (such as 8-OHdG as a marker of DNA oxidation), and cross-linking to proteins are the effects of DNA oxidation by ROS [29,30].

## 3. Role of Oxidative Stress via Genetics Causes in Infertility

Genetic aberrations associated with infertility range from chromosomal anomalies to epigenetic changes. Based on cumulative evidence, oxidative stress is known to cause infertility via the induction of changes in telomere length [29,30,31,32], nuclear abnormalities, DNA fragmentation [31], apoptosis [33], mutation [34], alteration in miRNAs, DNA methylation, histone modification, RNA regulation, DNA repair, transcription, protein degradation, and change in the expression of related genes [35,36,37,38,39]. Arsenic at moderate concentrations induces cell cycle arrest and apoptosis by modulating genome-wide gene expression, leading to compromised DNA repair and increased genome instability [36].

## 4. Arsenic and Oxidative Stress

Arsenic is a metalloid in water, soil, and air that exists in two forms: inorganic (iAs) and organic. Inorganic forms of arsenic are trivalent arsenite (As^3+^) and pentavalent arsenate (As^5+^), with the former being a more toxic form than the latter. Contaminated drinking water is the most important route of exposure for iAs. Arsenic produces ROS; the oxidation of arsenite to arsenate through arsenic methylation leads to OS and can cause damage to physiological functions of the cell leading to various diseases such as cancers, diabetes, atherosclerosis, and cardiovascular disease and infertility [12,40,41].

Some potential mechanisms of arsenic-induced OS are as follows:

- The alteration of mitochondrial integrity and membrane potential, the loss of mitochondrial organization, the release of cyt-c and activation of Bax (apoptotic protein), decreased expression of Bcl2, and apoptosis. Mitochondria produce ROS through complex I and III [42,43].
- The methylation of arsenic. The detoxification of arsenic is associated with its methylation in the liver by As3MT, and the production of its methylated metabolites include MMA^V^, MMA^III^, DMA^V^, and DMA^III^. In this pathway, arsenic needs glutathione (GSH) and other thiols. Depleting GSH and other thiols alters the redox status, producing arsenic methylated metabolites that increase oxidative stress [44,45,46].
- The alteration of some signaling pathways: such as the tyrosine phosphorylation pathway and mitogen-activated protein kinase (MAPK) pathway, and transcription factors such as NF-kB, AP-1, apoptosis, the activation of p53, and Bax expression [47].
- Damage to proteins, carbohydrates, lipids, and DNA. Arsenic causes damage to protein by producing •OH or O2•- that leads to the production of carbonyl, aldehydes, and keto compounds. This metalloid also damages some amino acid residues such as cysteine and methionine, and this may lead to alterations in protein structure, degradation, unfolding, fragmentation, the inactivation of enzymes (such as antioxidant enzymes, pyruvate dehydrogenase), and the production of advanced glycation end products (AGEs) [27].

Arsenic causes damage to carbohydrates leading to the production of ketoamines and ketoaldehydes, as well as changes in the carbohydrate metabolism (i.e., the inhibition of pyruvate dehydrogenase complex, hyperglycemia, and glucose intolerance) [48].

Arsenic causes damage to lipids leading to the production of fatty acid radicals (ROO•), MDA, HNE, the oxidation of cellular membranes, and the inactivation of membrane-bound receptors [49,50].

Arsenic may damage DNA, leading to alterations in DNA bases (such as the production of 8-OHdG; the altered bases can modify the site of binding of transcription factors and change the expression of related genes), alterations in DNA repair enzymes, DNA strand break, and the cross-linkage of DNA–protein [50].

## 5. Infertility and Oxidative Stress

There is limited information available about oxidative stress and the reproductive system. In normal conditions, a low level of ROS is essential for fertility [51]. Both spermatozoa and oocytes produce ROS that is essential for sperm–oocyte interaction. The production of ROS is balanced by the antioxidants. The excessive production of ROS impairs the function of spermatozoa and leads to DNA damage, sperm–oocyte fusion, and infertility [52,53]. A low level of H_2_O_2_ stimulates the function of sperm, such as the hyperactivity and interaction of sperm–oocyte [54]. NO has been found to play a vital role in folliculogenesis. A low level of NO is needed for the maturation of oocytes and fertilization. Some studies have shown that a high level of NO is associated with lower pregnancy rates [23]. Two important ROS production sources in the male reproductive systems are immature spermatozoa and seminal leukocytes [55]. Polymorphonuclear leukocytes (PMN), and macrophages produce 50–60% and 20–30% of ROS, respectively [56]. The activation of leukocytes by inflammation and infection produces higher ROS [57]. They increase the production of NADPH via the hexose monophosphate pathway and myeloperoxidase system, leading to ROS overproduction [57]. Two systems for producing ROS by spermatozoa are the NADPH oxidase system in the plasma membrane of sperm and the NADPH-dependent oxidoreductase in the mitochondria [58,59].

One of the causes of sperm dysfunction is oxidative stress. ROS may cause infertility by two mechanisms: (i) damage to the sperm membrane and the reduction in sperm motility, and (ii) damage to sperm DNA [60].

Spermatocytes are susceptible to oxidative stress-induced damage for high levels of polyunsaturated fatty acids (PUFA) in the plasma membrane. Lipid peroxidation is the most important cause of a loss of sperm motility [61]. Low levels of antioxidant enzymes such as catalase or glutathione (GSH) cause damage to spermatocytes [62].

Several biological mechanisms of the effects of oxidative stress on the functions of sperm cells have been identified. These include lipid peroxidation, the production of lipid hydroperoxides, interaction with transitional metals such as iron or copper, as well as the production of cytotoxic peroxyl radicals such as MDA and HNE that damage the membrane and function of sperm cells and sperm–oocyte fusion [23], DNA damage by the overproduction of ROS, the modification of bases such as 8-OHdG as a biomarker of oxidative DNA damage, chromosomal rearrangement, single- and double-strand DNA breaks, and a gene mutation that leads to the reduction in semen quality [29,30]. The excessive production of ROS in mitochondria leads to the release of apoptosis-inducing factors (AIF) and DNA fragmentation [63]. Damage to the mitochondrial membrane leads to the release of cyto-c and apoptosis. Phosphatidylserine as a marker of apoptosis is higher in the infertile patient than in normal men [33]. Varicocele is another cause of male infertility. Increased NO and the production of O2•- and 8-OHdG have been demonstrated in a patient with varicoceles. Smoking also reduces total sperm count, sperm density, and the number of motile sperm [24,64].

The main sources of ROS in the ovary are phagocytic macrophages, parenchymal steroidogenic cells, and endothelial cells [65]. Based on the role of ROS in folliculogenesis, the maturation of follicles, and ovulation, steroidogenic cells have potent antioxidant enzyme activity [66]. Low levels of some antioxidant enzymes in the follicular fluid, such as GPx, high levels of NO, and increased MDA have been observed in infertile women [67].

## 6. Arsenic Toxicity and Male and Female Reproductive Systems

**Figure 1:**
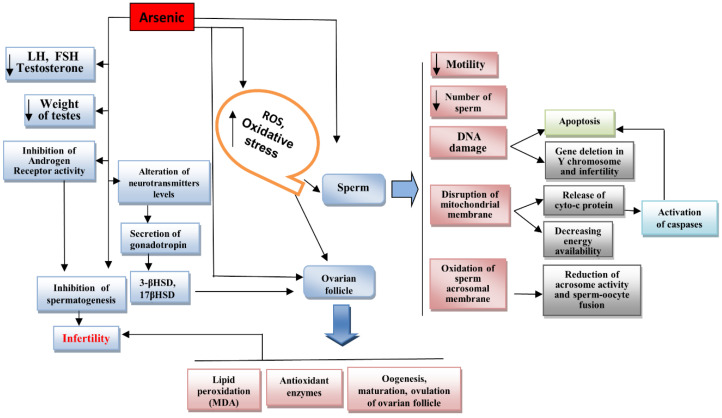
Effects of arsenic on the male and female reproductive systems.

Many studies have shown that arsenic is a potent toxicant to the reproductive system [68,69,70,71]. The male reproductive system is more susceptible to arsenic toxicity because arsenic can directly bind to SH-groups of proteins such as sperm chromatin and flagellum [71]. Arsenic also alters reproductive hormones and biogenic amines that regulate spermatogenesis and oogenesis [72]. Several negative effects of arsenic on the male and female reproductive systems are shown in Figure 1 and summarized in Table 1.

Arsenic exposure reduces the number of sperm due to reduced GSH and increased MDA [73]. It is further known to increase ROS levels in testes [73], alter the hormone secretion (i.e., reduction in testosterone, FSH, LH) [72,74], and disrupt spermatogenesis by inhibition of androgen receptor activity [75]. Arsenic interacts with cysteine residues in DNA-binding domain (DBD) of steroid receptors and inhibits their activity [76]. Arsenic exposure is also responsible for the reduction in testicular weight [9,77] and alterations in enzymes such as lactate dehydrogenase (LDH), acid phosphatase (ACP), γ-glutamyl transpeptidase (GGT) [9] as shown in Table 1.

Arsenic exposure reduced sperm motility and viability [9,77] and decreased the expression levels of CYP11A1 and CYP17A1 [37]. It also impaired sperm acrosome membrane protein 1 (SPACA1) and altered the shape of the sperm head [9,77], decreased VDAC3, and disturbed the fertilization process [11]. Arsenic exposure induced inflammation in the testes and increased the production of inflammatory factors such as TNF-α, COX, NF-kB, and caspase 3 [78].

Arsenic affects the female reproductive system through the alteration of some regulator enzymes in steroidogenesis such as 3β-hydroxysteroid dehydrogenase (3-βHSD) and 17β-hydroxysteroid dehydrogenase (17βHSD) due to low levels of gonadotropin [77,78,79,80], and a reduction in gonadotropin secretion due to alterations in the levels of some neurotransmitters (i.e., reduction in LH, FSH, and estradiol) [77,81].

## 7. Conclusions

Based on this review, arsenic with a direct effect or the induction of oxidative stress leads to dysfunction of the reproductive system. Further studies are necessary in order to clarify the proper molecular mechanisms involved in arsenic toxicity in the reproductive system. Several important preventive strategies to tackle this public health problem, especially in people exposed to arsenic, include: (i) the use of safe water (removal of arsenic in the drinking water), (ii) the consumption of a protein-rich diet (a diet rich in protein has a protective effect on arsenic-related toxicity), (iii) the use of antioxidant supplements such as vitamin C, E, and polyphenols, and probiotics for activation of the body defense system, (iv) modification in rice cooking methods (rice is the food source of arsenic), (v) maintaining a healthy lifestyle (physical regulatory activity, abstinence from alcohol consumption and smoking).

## Figures and Tables

**Table 1:** Various negative effects of arsenic on the male and female reproductive systems.

Negative Effects of Arsenic on the Male and Female Reproductive Systems	References
Reduction in the number of sperm (due to reduction in GSH and increased MDA)	[73]
Increase in the levels of ROS in testes	[73]
Alteration in hormone secretion (reduction in testosterone, FSH, LH)	[72,74]
Disruption of spermatogenesis by inhibition of androgen receptor activity	[75]
Interaction with the cysteine residues in DNA-binding domain (DBD) of steroid receptors inhibits their activity	[76]
Reduction in testicular weight	[9,77]
Alteration of some enzymes such as lactate dehydrogenase (LDH), acid phosphatase (ACP), γ-glutamyl transpeptidase (GGT)	[9]
Reduction in sperm motility and viability	[9,77]
Decrease in the expression level of CYP11A1, CYP17A1	[37]
Impaired sperm acrosome membrane protein 1 (SPACA1) and alteration in shape of sperm head	[9,77]
Decrease in VDAC3 and disturbance of fertilization process	[11]
Induction of inflammation in testes and increase in the production of inflammatory factors such as TNF-α, COX, NF-kB, caspase 3	[78]
Alteration of some regulator enzymes in steroidogenesis such as 3β-hydroxysteroid dehydrogenase (3-βHSD), 17β-hydroxysteroid dehydrogenase (17βHSD) due to low levels of gonadotropin	[77,78,79,80]
Reduction in gonadotropin secretion due to alteration of the levels of some neurotransmitters (reduction in LH, FSH, estradiol)	[77,81]

## Abbreviation

RNS reactive nitrogen species; ROS reactive oxygen species; FSH follicle-stimulating hormone; LH luteinizing hormone; OS oxidative Stress; MAO monoamine oxidase; XO xanthine oxidase; COX cyclooxygenase; LOX lipoxygenase; MPO myeloperoxidase; NOS nitric oxide synthase; NF-kB nuclear factor kappa B; NO nitric oxide; MDA malondialdehyde; HNE hydroxynonenal; GSH glutathione; MAPK mitogen-activated protein kinase; AGEs advanced glycation end products; PMN polymorphonuclear leukocytes; PUFA polyunsaturated fatty acids; AIF apoptosis inducing factor; DBD DNA-binding domain; LDH lactate dehydrogenase; ACP acid phosphatase; GGT γ-glutamyl transpeptidase; SPACA1 sperm acrosome membrane protein 1; VDAC3 voltage-dependent anion channel protein 3; TNF-α tumor necrosis factor.

## References

[1] World Health Organization (2021). WHO fact sheet on infertility. Glob. Reprod. Health.

[2] Practice Committee of the American Society for Reproductive Medicine (2020). Evidence-based treatments for couples with unexplained infertility: A guideline. Fertil. Steril..

[3] Briffa J., Sinagra E., Blundell R. (2020). Heavy metal pollution in the environment and their toxicological effects on humans. Heliyon.

[4] Wang X., Zhang J., Xu W., Huang Q., Liu L., Tian M., Xia Y., Zhang W., Shen H. (2016). Low-level environmental arsenic exposure correlates with unexplained male infertility risk. Sci. Total Environ..

[5] Erkan M., Aydin Y., Yilmaz B.O., Yildizbayrak N. (2021). Arsenic-induced oxidative stress in reproductive systems. Toxicology.

[6] Rami Y., Ebrahimpour K., Maghami M., Shoshtari-Yeganeh B., Kelishadi R. (2022). The Association Between Heavy Metals Exposure and Sex Hormones: A Systematic Review on Current Evidence. Biol. Trace Elem. Res..

[7] Pan W., Ye X., Zhu Z., Li C., Zhou J., Liu J. (2020). A case-control study of arsenic exposure with the risk of primary ovarian insufficiency in women. Environ. Sci. Pollut. Res..

[8] Lei H.L., Wei H.J., Ho H.Y., Liao K.W., Chien L.C. (2015). Relationship between risk factors for infertility in women and lead, cadmium, and arsenic blood levels: A cross-sectional study from Taiwan. BMC Public Health.

[9] Renu K., Madhyastha H., Madhyastha R., Maruyama M., Vinayagam S., Gopalakrishnan A.V. (2018). Review on molecular and biochemical insights of arsenic-mediated male reproductive toxicity. Life Sci..

[10] Barsøe I.M., Ebdrup N.H., Clausen H.S., Lyngsø J., Schullehner J., Ramlau-Hansen C.H., Knudsen U.B. (2021). Drinking Water Arsenic and Adverse Reproductive Outcomes in Men and Women: A Systematic PRISMA Review. Water.

[11] Huang Q., Luo L., Alamdar A., Zhang J., Liu L., Tian M., Eqani S.A., Shen H. (2016). Integrated proteomics and metabolomics analysis of rat testis: Mechanism of arsenic-induced male reproductive toxicity. Sci. Rep..

[12] Wai K.M., Umezaki M., Mar O., Umemura M., Watanabe C. (2019). Arsenic exposure through drinking Water and oxidative stress Status: A cross-sectional study in the Ayeyarwady region, Myanmar. J. Trace Elem. Med. Biol..

[13] Yaribeygi H., Sathyapalan T., Atkin S.L., Sahebkar A. (2020). Molecular mechanisms linking oxidative stress and diabetes mellitus. Oxid Med. Cell Longev..

[14] Khosravi M., Poursaleh A., Ghasempour G., Farhad S., Najafi M. (2019). The.effects of oxidative stress on the development of atherosclerosis. Biol. Chem..

[15] Hayes J.D., Dinkova-Kostova A.T., Tew K.D. (2020). Oxidative stress in cancer. Cancer Cell.

[16] Aitken R.J. (2020). Impact of oxidative stress on male and female germ cells: Implications for fertility. Reproduction.

[17] Sarmiento-Salinas F.L., Perez-Gonzalez A., Acosta-Casique A., Ix-Ballote A., Diaz A., Treviño S., Maycotte P. (2021). Reactive oxygen species: Role in carcinogenesis, cancer cell signaling and tumor progression. Life Sci..

[18] Klaunig J.E. (2018). Oxidative stress and cancer. Curr. Pharm. Des..

[19] Hoque S.M., Umehara T., Kawai T., Shimada M. (2021). Adverse effect of superoxide-induced mitochondrial damage in granulosa cells on follicular development in mouse ovaries. Free Radic. Biol. Med..

[20] Milkovic L., Cipak Gasparovic A., Cindric M., Mouthuy P.A., Zarkovic N. (2019). Short overview of ROS as cell function regulators and their implications in therapy concepts. Cells.

[21] Rendra E., Riabov V., Mossel D.M., Sevastyanova T., Harmsen M.C., Kzhyshkowska J. (2019). Reactive oxygen species (ROS) in macrophage activation and function in diabetes. Immunobiology.

[22] Latchoumycandane C., Vaithinathan S., D’Cruz S.C., Mathur P.P. (2020). Apoptosis and male infertility. Male Infertility.

[23] Dutta S., Sengupta P. (2022). The Role of Nitric Oxide on Male and Female Reproduction. Malays. J. Med. Sci. MJMS.

[24] Lorian K., Kadkhodaee M., Kianian F., Abdi A., Sadeghipour H., Seifi B. (2019). Oxidative stress, nitric oxide and inflammation in the pathophysiology of varicocele and the effect of hydrogen sulfide as a potential treatment. J. Physiol. Pharmacol..

[25] Pujianto D.A., Oktarina M., Sharma Sharaswati I.A., Yulhasri (2021). Hydrogen peroxide has adverse effects on human sperm quality parameters, induces apoptosis, and reduces survival. J. Hum. Reprod. Sci..

[26] Peña F.J., O’Flaherty C., Ortiz Rodríguez J.M., Martín Cano F.E., Gaitskell-Phillips G.L., Gil M.C., Ortega Ferrusola C. (2019). Redox regulation and oxidative stress: The particular case of the stallion spermatozoa. Antioxidants.

[27] Zargari F., Kumar N. (2021). Arsenic and Oxidative Stress: An Overview. Arsenic Toxicity: Challenges and Solutions.

[28] Barrera G., Pizzimenti S., Daga M., Dianzani C., Arcaro A., Cetrangolo G.P., Gentile F. (2018). Lipid peroxidation-derived aldehydes, 4-hydroxynonenal and malondialdehyde in aging-related disorders. Antioxidants.

[29] Bui A.D., Sharma R., Henkel R., Agarwal A. (2018). Reactive oxygen species impact on sperm DNA and its role in male infertility. Andrologia.

[30] Roy B. (2018). Physiology of stress and the involvement of reactive oxidative species: A mini-review. Quest Int. J. Med. Health Sci..

[31] Berby B., Bichara C., Rives-Feraille A., Jumeau F., Pizio P.D., Sétif V., Sibert L., Dumont L., Rondanino C., Rives N. (2021). Oxidative Stress Is Associated with Telomere Interaction Impairment and Chromatin Condensation Defects in Spermatozoa of Infertile Males. Antioxidants.

[32] Rocca M.S., Foresta C., Ferlin A. (2019). Telomere length: Lights and shadows on their role in human reproduction. Biol. Reprod..

[33] Asadi A., Ghahremani R., Abdolmaleki A., Rajaei F. (2021). Role of sperm apoptosis and oxidative stress in male infertility: A narrative review. Int. J. Reprod. Biomed..

[34] Aitken R.J., Baker M.A. (2020). The Role of Genetics and Oxidative Stress in the Etiology of Male Infertility—A Unifying Hypothesis?. Front. Endocrinol..

[35] Hu Y., Li J., Lou B., Wu R., Wang G., Lu C., Wang H., Pi J., Xu Y. (2020). The Role of Reactive Oxygen Species in Arsenic Toxicity. Biomolecules.

[36] Kim D., Park N.Y., Kang K., Calderwood S.K., Cho D.H., Bae I.J., Bunch H. (2021). Arsenic hexoxide has differential effects on cell proliferation and genome-wide gene expression in human primary mammary epithelial and MCF7 cells. Sci. Rep..

[37] Taşçı T., Eldem V., Erkan M. (2019). Sodium Arsenic Alters the Gene Expression of some Steroidogenic Genes in TM3 Leydig Cell. Celal Bayar Univ. J. Sci..

[38] Eckstein M., Eleazer R., Rea M., Fondufe-Mittendorf Y. (2017). Epigenomic reprogramming in inorganic arsenic-mediated gene expression patterns during carcinogenesis. Rev. Environ. Health.

[39] Rzymski P., Tomczyk K., Rzymski P., Poniedziałek B., Opala T., Wilczak M. (2015). Impact of heavy metals on the female reproductive system. Ann. Agric. Environ. Med..

[40] Palma-Lara I., Martínez-Castillo M., Quintana-Pérez J.C., Arellano-Mendoza M.G., Tamay-Cach F., Valenzuela-Limón O.L., Hernández-Zavala A. (2020). Arsenic exposure: A public health problem leading to several cancers. Regul. Toxicol. Pharmac..

[41] Minatel B.C., Sage A.P., Anderson C., Hubaux R., Marshall E.A., Lam W.L., Martinez V.D. (2018). Environmental arsenic exposure: From genetic susceptibility to pathogenesis. Environ. Int..

[42] Muller F.L., Liu Y., Van Remmen H. (2004). Complex III releases superoxide to both sides of the inner mitochondrial membrane. J. Biol. Chem..

[43] Mishra D., Mehta A., Flora S.J. (2008). Reversal of arsenic-induced hepatic apoptosis with combined administration of DMSA and its analogues in guinea pigs: Role of glutathione and linked enzymes. Chem. Res. Toxicol..

[44] Németi B., Gregus Z. (2002). Reduction of arsenate to arsenite in hepatic cytosol. Toxicol. Sci..

[45] Dopp E., Kligerman A.D., Diaz-Bone R.A. (2010). Organoarsenicals. Uptake, metabolism, and toxicity. Met. Ions Life Sci..

[46] Thomas D.J., Li J., Waters S.B., Xing W., Adair B.M., Drobna Z., Devesa V., Styblo M. (2007). Arsenic (+3 oxidation state) methyltransferase and the methylation of arsenicals. Exp. Biol. Med..

[47] Nagesh R., Kiran Kumar K.M., Naveen Kumar M., Patil R.H., Sharma S.C. (2019). Stress activated p38 MAPK regulates cell cycle via AP-1 factors in areca extract exposed human lung epithelial cells. Cytotechnology.

[48] Sabir S., Akash M.S.H., Fiayyaz F., Saleem U., Mehmood M.H., Rehman K. (2019). Role of cadmium and arsenic as endocrine disruptors in the metabolism of carbohydrates: Inserting the association into perspectives. Biomed. Pharmacother..

[49] Wirtitsch M., Roth E., Bachleitner-Hofmann T., Wessner B., Sturlan S. (2009). Omega-3 and omega-6 polyunsaturated fatty acids enhance arsenic trioxide efficacy in arsenic trioxide-resistant leukemic and solid tumor cells. Oncol. Res..

[50] De Vizcaya-Ruiz A., Barbier O., Ruiz-Ramos R., Cebrian M.E. (2009). Biomarkers of oxidative stress and damage in human populations exposed to arsenic. Mutat. Res. Genet. Toxicol. Environ. Mutagen..

[51] Scarlata E., O’Flaherty C. (2020). Antioxidant enzymes and male fertility: Lessons from knockout models. Antioxid. Redox. Signal..

[52] Bartsch H., Nair J. (2004). Oxidative stress and lipid peroxidation-derived DNA-lesions in inflammation driven carcinogenesis. Cancer Detect. Prev..

[53] Agarwal A., Leisegang K., Sengupta P. (2020). Oxidative stress in pathologies of male reproductive disorders. Pathology.

[54] Durairajanayagam D. (2019). Physiological role of reactive oxygen species in male reproduction. Oxidants, Antioxidants and Impact of the Oxidative Status in Male Reproduction.

[55] Garrido N., Meseguer M., Simon C., Pellicer A., Remohi J. (2004). Pro-oxidative and anti-oxidative imbalance in human semen and its relation with male fertility. Asian J. Androl..

[56] Thomas J., Fishel S.B., Hall J.A., Green S., Newton T.A., Thornton S.J. (1997). Increased polymorphonuclear granulocytes in seminal plasma in relation to sperm morphology. Hum. Reprod..

[57] Agarwal A., Rana M., Qiu E., AlBunni H., Bui A.D., Henkel R. (2018). Role of oxidative stress, infection and inflammation in male infertility. Andrologia.

[58] Fatehi D., Moayeri A., Rostamzadeh O., Rostamzadeh A., Kebria M.M. (2018). Reactive oxygenated species (ROS) in male fertility; source, interaction mechanism and antioxidant therapy. Res. J. Pharm. Technol..

[59] Kumar N., Singh A.K. (2018). Reactive oxygen species in seminal plasma as a cause of male infertility. J. Gynecol. Obstet. Hum. Reprod..

[60] Agarwal A., Sharma R.K., Nallella K.P., Thomas A.J., Alvarez J.G., Sikka S.C. (2006). Reactive oxygen species as an independent marker of male factor infertility. Fertil. Steril..

[61] Nowicka-Bauer K., Nixon B. (2020). Molecular changes induced by oxidative stress that impair human sperm motility. Antioxidants.

[62] Nsonwu-Anyanwu A.C., Ekong E.R., Offor S.J., Awusha O.F., Orji O.C., Umoh E.I., Usoro C.A.O. (2019). Heavy metals, biomarkers of oxidative stress and changes in sperm function: A case-control study. Int. J. Reprod. Biomed..

[63] Ding L., Li J., Li W., Fang Z., Li N., Wu S., Hong M. (2018). p53-and ROS-mediated AIF pathway involved in TGEV-induced apoptosis. J. Vet. Med. Sci..

[64] Barati E., Nikzad H., Karimian M. (2020). Oxidative stress and male infertility: Current knowledge of pathophysiology and role of antioxidant therapy in disease management. Cell Mol. Life Sci..

[65] Terao H., Wada-Hiraike O., Nagumo A., Kunitomi C., Azhary J.M., Harada M., Osuga Y. (2019). Role of oxidative stress in follicular fluid on embryos of patients undergoing assisted reproductive technology treatment. J. Obstet. Gynaecol..

[66] Amano T., Chano T. Linking oxidative stress and ovarian cancers. Cancer.

[67] Mohammadi M. (2019). Oxidative stress and polycystic ovary syndrome: A brief review. Int. J. Prev. Med..

[68] Mehta M., Hundal S.S. (2016). Effect of sodium arsenite on reproductive organs of female Wistar rats. Arch. Environ. Occup. Health.

[69] Wirth J.J., Mijal R.S. (2010). Adverse effects of low level heavy metal exposure on male reproductive function. Syst. Biol. Reprod. Med..

[70] Kippler M., Wagatsuma Y., Rahman A., Nermell B., Persson L.Å., Raqib R., Vahter M. (2012). Environmental exposure to arsenic and cadmium during pregnancy and fetal size: A longitudinal study in rural Bangladesh. Reprod. Toxicol..

[71] De Palma G., Ortiz A., Apostoli P. (2022). Effects of metallic elements on reproduction and development. Handbook on the Toxicology of Metals.

[72] Jana K., Jana S., Samanta P.K. (2006). Effects of chronic exposure to sodium arsenite on hypothalamo-pituitary-testicular activities in adult rats: Possible an estrogenic mode of action. Reprod. Biol. Endocrinol..

[73] Im Chang S., Jin B., Youn P., Park C., Park J.D., Ryu D.Y. (2007). Arsenic-induced toxicity and the protective role of ascorbic acid in mouse testis. Toxicol. Appl. Pharmacol..

[74] Saberi Sis F., Zargari F. (2017). The effect of aqueous extract of white tea on serum levels of FSH, LH and testosterone in rats exposed to arsenic. J. Fasa Univ. Med. Sci..

[75] Rosenblatt A.E., Burnstein K.L. (2009). Inhibition of androgen receptor transcriptional activity as a novel mechanism of action of arsenic. J. Mol. Endocrinol..

[76] Kaltreider R.C., Davis A.M., Lariviere J.P., Hamilton J.W. (2001). Arsenic alters the function of the glucocorticoid receptor as a transcription factor. Environ. Health Perspect..

[77] Ilieva I., Sainova I., Yosifcheva K. (2021). Toxic Effects of Heavy Metals (Mercury and Arsenic) on the Male Fertility. Acta Morphol. Anthropol..

[78] Shao Y., Zhao H., Wang Y., Liu J., Li J., Chai H., Xing M. (2018). Arsenic and/or copper caused inflammatory response via activation of inducible nitric oxide synthase pathway and triggered heat shock protein responses in testis tissues of chicken. Environ. Sci. Pollut. Res. Int..

[79] Chattopadhyay S., Ghosh S., Chaki S., Debnath J., Ghosh D. (1999). Effect of sodium arsenite on plasma levels of gonadotrophins and ovarian steroidogenesis in mature albino rats: Duration-dependent response. J. Toxicol. Sci..

[80] Reddy V.B.M., Reddy P.S., Sasikala P., Reddy Y.V.K. (2010). Transplacental and lactational exposure of arsenic to mice: Effect on steroidogenic enzymes and hormones of male reproduction. Int. J. Toxicol. Pharmacol. Res..

[81] Bhardwaj J.K., Paliwal A., Saraf P. (2021). Effects of heavy metals on reproduction owing to infertility. J. Biochem. Mol. Toxicol..

